# Outcomes of Tiopronin and D-Penicillamine Therapy in Pediatric Cystinuria: A Clinical Comparison of Two Cases

**DOI:** 10.3390/reports8030163

**Published:** 2025-09-01

**Authors:** Brooke Schaefer, Adinoyi Garba, Xiaoyan Wu

**Affiliations:** 1 Department of Pediatrics, University at Buffalo, Buffalo, NY 14203, USA; bnschaef@buffalo.edu; 2 School of Pharmacy, D’Youville University, Buffalo, NY 14201, USA; garbaa@dyc.edu

**Keywords:** cystinuria, cystine, cysteine, nephrolithiasis, SLC7A9 mutation, tiopronin, D-penicillamine

## Abstract

**Background and Clinical Significance:** Cystinuria is the most common genetic cause of pediatric nephrolithiasis, characterized by impaired renal cystine reabsorption and resulting in increased urinary cystine excretion. Due to the poor solubility of cystine at normal urine pH, increased urinary cystine excretion leads to urine supersaturation and precipitation of cystine, resulting in nephrolithiasis. **Case Presentation:** Here, we report two cases of female patients diagnosed with cystinuria caused by *SLC7A9* mutations. Both patients were initially managed with conservative treatments to minimize stone recurrence including increased oral fluid intake, a low-salt/low-protein diet, and potassium citrate supplementation with the goal of reducing urinary cystine levels and minimizing stone recurrences. Due to persistent stone formation, the patients were started on two distinct cystine-binding thiol medications. One patient was initiated on tiopronin, and the other on D-penicillamine. Tiopronin and D-penicillamine are both used in the treatment of pediatric cystinuria, although tiopronin is often preferred due to its more favorable side effect profile. However, due to insurance constraints, D-penicillamine was initiated for one patient in place of tiopronin. Since the initiation of these two distinct cystine-binding thiol medications, both patients have demonstrated reduced urinary cystine excretion and minimal to no recurrence of kidney stones. **Conclusions:** Cystine-binding thiols, including tiopronin and D-penicillamine, can both be used in the management of cystinuria in pediatric patients.

## 1. Introduction and Clinical Significance

Nephrolithiasis, or kidney stone disease, is less common in children than in adults, but has shown a rising prevalence over the past several decades [1]. Population-based studies in the United States estimate the incidence of pediatric urolithiasis to be approximately 65 per 100,000 person-years during the 2005–2016 period—an increase from 18 per 100,000 person-years reported in 1999 [2].

Cystinuria accounts for approximately 1–2% of all kidney stones and 6–8% of pediatric kidney stones, making it a significant genetic cause of nephrolithiasis in children. Cystinuria is an autosomal recessive disorder caused by mutations in the SLC3A1 and/or SLC7A9 genes, which encode subunits of the amino acid transporter responsible for reabsorbing cystine and dibasic amino acids in the renal proximal tubule. Mutations in these genes lead to impaired tubular reabsorption, resulting in excess cystine excretion. Because cystine is poorly soluble in acidic urine, it readily precipitates and forms recurrent stones.

Among the two genes, mutations in SLC7A9 are often associated with a more variable but potentially more severe phenotype compared to SLC3A1 mutations, particularly in terms of earlier onset and higher stone burden in some patients [3]. Approximately 53% of individuals with cystinuria harbor mutations in SLC7A9 [4], though prevalence may vary by population and screening methodology. Genetic studies have also shown that SLC7A9 mutations may exhibit incomplete penetrance, contributing to a wider clinical spectrum, ranging from asymptomatic hyperexcretion of cystine to severe recurrent urolithiasis.

Treatment options for cystinuria include general kidney stone prevention approaches like proper hydration, low salt intake, and alkalinization of urine. Recent systematic reviews reaffirm that the combination of optimized diuresis, urinary alkalinization, and thiol therapy effectively reduces urinary cystine levels, cystine crystal formation, and stone recurrence in cystinuria management [5]. More targeted approaches for cystinuria include thiol-binding medications, such as tiopronin and D-penicillamine. These medications reduce cystine to two cysteine molecules, and form more soluble mixed disulfide complexes with the cysteine molecules. The thiol-cysteine compound is more soluble in urine, which in turn reduces cystine stone formation. Based on multiple clinical studies, tiopronin is considered the first-line thiol agent in pediatric cystinuria due to its favorable safety profile. Tiopronin has a significantly lower incidence of serious adverse effects, particularly nephrotic syndrome, bone marrow suppression, and severe dermatologic reactions [6]. Due to fewer gastrointestinal and systemic side effects, tiopronin is associated with better medication adherence in pediatric patients [7]. D-penicillamine has been used in pediatric patients with cystinuria for decades, with variable efficacy and a notable risk of adverse effects [8,9,10,11]. 

Here, we present two pediatric cases of cystinuria, both involving mutations in the SLC7A9 gene. Despite initial management with increased oral hydration and urine alkalinization, both patients experienced persistent stone formation. As a result, one patient was initiated on tiopronin and the other on D-penicillamine, due to insurance limitations. Urinary cystine excretion, renal function, and treatment-related side effects were closely monitored. Both patients demonstrated meaningful clinical improvement following thiol therapy.

## 2. Case Presentation

### 2.1. Case 1

A 14-year-old Caucasian female with a medical history of anxiety disorder managed with sertraline, bilateral vesicoureteral reflux, and a duplicated collecting system presented to the emergency department (ED) with intermittent abdominal pain, left flank pain, and generalized weakness.

A non-contrast CT scan revealed 2 mm right ureterovesical junction (UVJ) stone and punctate, non-obstructing bilateral renal calculi. Laboratory evaluation included a complete blood count (CBC) and basic metabolic panel (serum sodium, potassium, chloride, bicarbonate, blood urea nitrogen, creatinine, and glucose) were within normal limits, and urinalysis showed no evidence of infection. She was started on tamsulosin and nonsteroidal anti-inflammatory drugs (NSAIDs) for pain per urology recommendations and discharged with instructions to attempt spontaneous stone passage. She was subsequently referred to nephrology for further evaluation. Review of systems revealed a positive family history of nephrolithiasis on her father’s side.

**Figure 1:**
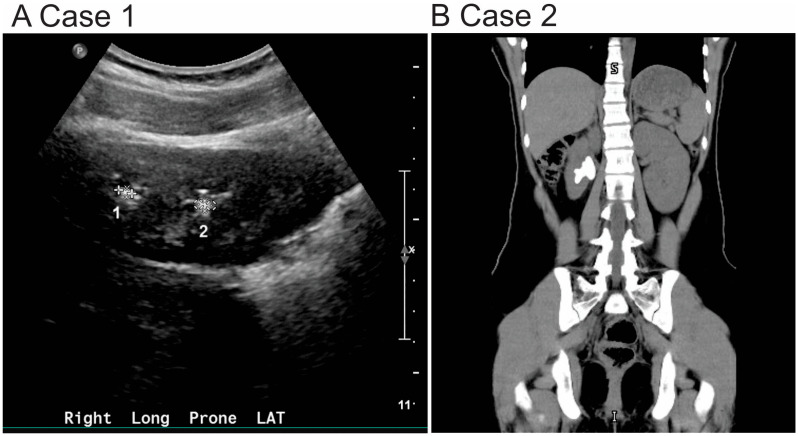
Radiographic findings in two pediatric patients diagnosed with cystinuria. (**A**) Renal ultrasound of Case 1: The kidneys are normal in size and position. In the right kidney, there are subcentimeter calculi at the upper and lower poles, each measuring up to 4 mm (indicated as 1 and 2). In the left kidney, a 3 mm nonobstructing calculus is seen at the lower pole (data not shown). No hydronephrosis or hydroureter is present. (**B**) Non-contrast CT of the abdomen and pelvis (Case 2): A 3.5 cm staghorn calculus is seen extending from the mid to lower pole of the right kidney into the renal pelvis. No ureteral or left-sided calculi are identified.

In the renal clinic, her serum creatinine and kidney function were within normal limits. However, 24 h urine studies showed hypercalciuria and hypocitraturia. Potassium citrate therapy was initiated along with recommendations for increased oral fluid intake and dietary sodium restriction. Despite these measures, the patient continued to experience symptoms and underwent two surgical interventions for left ureterolithiasis and left ureteral calculi. Follow-up renal ultrasound showed persistent non-obstructing renal stones measuring 3–4 mm (Figure 1A).

Given the failure of conservative management, genetic testing was pursued, which revealed a pathogenic mutation in the SLC7A9 gene. Her 24 h urinary cystine excretion was markedly elevated at 525 μmol/24 h (126 mg/24 h; calculated using μmol × 0.24 = mg), exceeding the normal reference range of 17–102 μmol/24 h. These findings confirmed the diagnosis of cystinuria (Table 1).

The patient was initiated on tiopronin at a dose of 200 mg, three times per day. She initially reported mild side effects, including nausea, fatigue, and muscle weakness. After one month of therapy, her 24 h urinary cystine excretion remained elevated at 921 μmol/24 h (221 mg/24 h). Consequently, the tiopronin dose was increased to 300 mg three times per day (Table 1).

During the first year of tiopronin therapy, the patient required hospitalization for a total of four urological procedures including: cystoscopy, right ureteral stent placement, right retrograde pyelogram and right ureteroscopy with stent removal. These interventions resulted in successful removal of preformed stones. Notably, no further surgical procedures were required after the first year of treatment of tiopronin.

**Figure 2:**
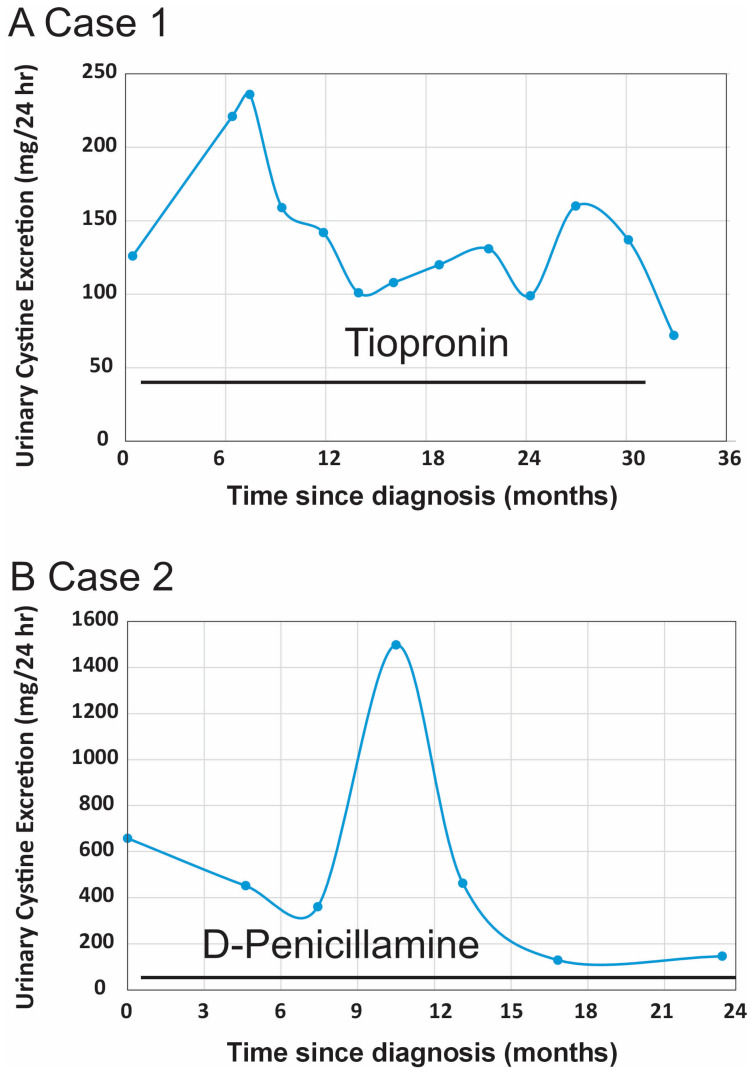
Longitudinal urinary cystine excretion in two pediatric patients with cystinuria due to *SLC7A9* mutations. The therapeutic goal for cystinuria management is to maintain urinary cystine excretion below 200 mg/L. (**A**) Case 1 (on Tiopronin) demonstrated a reduction in 24 h urinary cystine excretion and acceptable cystine supersaturation levels following initiation of Tiopronin. (**B**) Case 2 (on D-penicillamine) showed a downward trend in 24 h urinary cystine excretion after starting D-penicillamine. A temporary peak in cystine levels occurred during international travel when the patient had limited access to medication.

The patient remained on adjunctive therapy with potassium citrate 10 mEq twice daily. She was monitored in the renal clinic every three months. 24 h urinary cystine excretion, cystine supersaturation, and cystine capacity were assessed. These values have remained within the normal range throughout the follow-up period (Table 2). A longitudinal assessment of her urinary cystine excretion over an approximately three-year period is shown in Figure 2A, Case 1.

### 2.2. Case 2

An 11-year-old female initially presented to the emergency department (ED) with right-sided flank pain. A non-contrast CT scan revealed renal calculus, which she spontaneously passed two days later. She reported a history of intermittent left-sided flank pain since age seven but denied dysuria or hematuria.

She was subsequently evaluated in urology after being discharged from the ED. A repeat non-contrast CT imaging of the abdomen and pelvis revealed a 3.5 cm staghorn calculus occupying the mid to lower pole of the right kidney, extending into the renal pelvis (Figure 1B). Her basic metabolic panel and other laboratory studies were within normal limits. Stone analysis determined 100% cystine composition. She was referred to nephrology for further evaluation.

Conservative treatment for kidney stone management was initiated including potassium citrate 15 mEq twice daily, increased oral fluid intake, and dietary sodium restriction. Despite these measures, she experienced recurrent nephrolithiasis and ultimately required four endourological interventions including: cystoscopy, right ureteral stent placement, and right percutaneous nephrolithotomy for stone burdens > 2 cm.

Due to recurrent nephrolithiasis while on conservative treatment, genetic testing was pursued and revealed a homozygous pathogenic variant in the *SLC7A9* gene, confirming a diagnosis of cystinuria (Table 1). Pharmacologic therapy with tiopronin was recommended; however, despite multiple appeals, insurance authorization for tiopronin was denied. As an alternative, she started on D-penicillamine 250 mg once daily, which was subsequently titrated to 250 mg twice daily. The patient tolerated D-penicillamine well until the family independently financed extended international travel to visit relative abroad. During this time, a temporary interruption in medication availability led to a transient increase in urinary cystine excretion, which normalized after re-initiation of D-penicillamine upon returning to the United States.

The patient was prescribed potassium citrate 10 mEq twice daily. This relatively conservative dose was chosen based on her age, body weight, and tolerance, and was intended primarily to optimize urinary citrate levels and reduce stone risk. She continued potassium citrate 10 mEq twice daily throughout treatment.

Because D-penicillamine may interfere with vitamin B6 metabolism [12], she started pyridoxine supplementation. Her vitamin B6 levels have remained within the normal range during therapy. The patient has denied hematuria, abdominal or back pain, nausea, vomiting, diarrhea, or other adverse effects while on D-penicillamine.

While receiving D-penicillamine therapy, the patient required one percutaneous nephrolithotomy and two ureteral stent placements, each followed by stent removal. Ongoing monitoring has included serial assessments of 24 h urinary cystine excretion, renal function, and surveillance for potential adverse effects of D-penicillamine. A transient increase in urinary cystine excretion was noted during a period of international travel, when the patient experienced a temporary interruption in medication availability. Following her return to the United States and re-initiation of D-penicillamine, cystine levels returned to the target range. Her urinary cystine excretion values and cystine supersaturation data are summarized in Table 2. A longitudinal assessment of her 24 h urinary cystine levels over a 2-year period is shown in Figure 2B (Case 2).

The 24 h urine volumes in our patients ranged from 0.4 L to nearly 4 L, which is outside the typical reported average of 1.76 +/− 0.7 L [13]. These differences likely reflect variations in hydration status, adherence to fluid recommendations, and collection timing. Such variability emphasizes the challenge of maintaining consistent high fluid intake in pediatric patients with cystinuria.

## 3. Discussion

These two pediatric cases of cystinuria illustrate the phenotypic variability and management challenges associated with SLC7A9 mutations. While both patients shared the same genetic etiology, the magnitude of stone burden, urinary cystine excretion, and treatment accessibility varied significantly. The comparison of these two cases emphasizes the unpredictable clinical expression of cystinuria, even within genetically similar individuals (Table 1).

Case 1 is a female patient diagnosed with cystinuria at age 17 after experiencing recurrent nephrolithiasis, which required two surgical interventions. At the time of diagnosis, her 24 h urinary cystine excretion was 126 mg/24 h (138 mg/1.73 m^2^/24 h), markedly exceeding the diagnostic threshold of <48 mg/1.73 m^2^/24 h. She was initiated on tiopronin at 11 mg/kg/day, later titrated to 16 mg/kg/day in three divided doses (Table 1). The treatment was well tolerated. Both cystine supersaturation and cystine capacity remained within the target range (Table 2), and her urinary cystine concentrations consistently stayed below the therapeutic target of 200 mg/L (Figure 2A, Case 1). Notably, no further surgical interventions were required after the first year of therapy.

Case 2 is also a female patient diagnosed with cystinuria at age 15, following recurrent nephrolithiasis, including a 3.5 cm staghorn calculus. She underwent four endourological procedures for nephrolithiasis management before the diagnosis of cystinuria was made. Her urinary cystine excretion at the time of diagnosis was markedly elevated at 658 mg/24 h (807 mg/1.73 m^2^/24 h). Due to insurance denial of tiopronin, she was started on D-penicillamine at 33 mg/kg/day in three divided doses (Table 1). Although this drug is associated with a higher risk of adverse effects, the patient tolerated it well with vitamin B6 supplementation and close monitoring. Urinary cystine levels were reduced to target range (Figure 2B, Case 2), and the patient remained free of significant side effects or new stone formation during follow-up.

These cases illustrate key differences in the safety and tolerability profiles of thiol-based therapies. Tiopronin is widely regarded as the preferred first-line agent due to its improved side effect profile and ease of titration. Common side effects of tiopronin include gastrointestinal discomfort, proteinuria, and hematologic abnormalities, though these tend to be less frequent and severe. D-penicillamine is linked to a broader range of side effects such as rash, leukopenia, thrombocytopenia, nephrotic syndrome, and pyridoxine deficiency [4,12]. Nevertheless, as shown in Case 2, D-penicillamine remains an effective second-line option when tiopronin is inaccessible, provided that careful monitoring is in place.

The mechanism of action for both drugs involves cleaving the disulfide bond in cystine to form two cysteine molecules. Both drugs form a compound with the cysteine molecules, which is more soluble in urine than cystine. This helps maintain cystine concentrations below the solubility threshold of 200–250 mg/L, thereby reducing the risk of stone formation. Regular monitoring of 24 h urinary cystine excretion, urinary supersaturation, and cystine capacity, along with imaging surveillance, is essential to guide treatment efficacy and prevent recurrence [8].

Although stone composition analysis was not performed on retrieved fragments in our cases, it is likely that cystine constituted in the nidus, based on the patients’ known cystinuria and radiographic appearance. Secondary deposition of calcium salts may also have contributed to more complex morphologies, such as staghorn calculi, as described in prior studies. This limitation highlights the need for systematic stone analysis to better understand stone heterogeneity in cystinuria.

**Figure 3:**
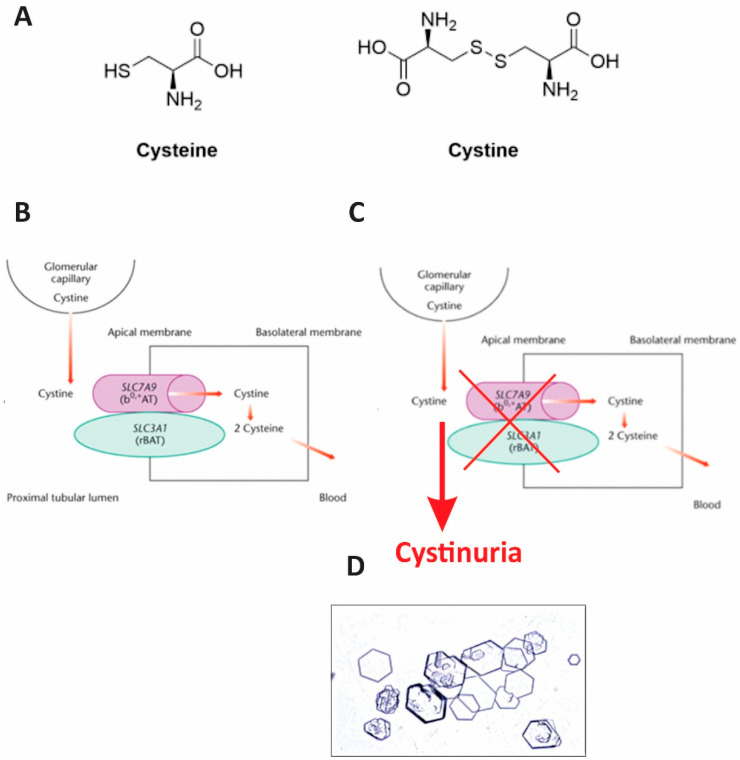
Pathogenesis of cystinuria. (**A**) Cystine is a dimer of two cysteine molecules linked by a disulfide bond. (**B**) Under physiological conditions, cystine is freely filtered by the glomerulus and reabsorbed in the proximal tubule (**left**). (**C**) In cystinuria, defective tubular transporters result in impaired reabsorption, leading to elevated urinary cystine levels and supersaturation. In the distal tubule, this supersaturation promotes cystine precipitation and stone (calculi) formation, which can subsequently damage renal structures. (**D**) Microscopic image of urinary cystine crystals from the patient with cystinuria. The image was captured through the eyepiece of a light microscope after centrifugation of a urine sample and dissolution of the cystine crystals. Characteristic hexagonal cystine crystals are visible, consistent with the diagnosis of cystinuria.

Pathogenesis of cystinuria is described in Figure 3. Under normal physiology, cystine is reabsorbed in the proximal tubule via the heterodimeric transporter composed of b(0,+)AT (encoded by SLC7A9) and rBAT (encoded by SLC3A1). Mutations in either gene impair reabsorption, leading to hyperexcretion of cystine and other dibasic amino acids in the urine. The poor solubility of cystine in acidic urine promotes crystal formation and stone development. Both patients in our report carried SLC7A9 mutations, leading to defective cystine transport and clinical manifestations of cystinuria.

Imaging plays a crucial role in diagnosis and follow-up. CT scans are highly sensitive and provide detailed assessment in these cases, particularly for complex stones like staghorn calculi. Renal ultrasound remains the first-line imaging modality in children due to its safety and absence of ionizing radiation [14].

The diagnosis of cystinuria is confirmed by a combination of clinical history, 24 h urine analysis showing cystine excretion > 48 mg/1.73 m^2^/day or >120–135 mg/g creatinine, stone composition analysis, and genetic testing. In both patients, identification of SLC7A9 mutations provided genetic confirmation, guided treatment decisions, and offered important insights for family counseling.

Our cases underscore the critical importance of maintaining consistent thiol therapy, as recent evidence shows that disruptions—such as during the COVID-19 pandemic—can lead to renal deterioration and stone progression in pediatric cystinuria patients [15]. Emerging evidence suggests that SGLT2 inhibitors like dapagliflozin may reduce stone recurrence and stabilize stone burden, indicating promising adjunctive or alternative strategies beyond thiol-based treatments [16]. Finally, patients on tiopronin have been shown to experience better health-related quality of life, and cystine capacity is gaining traction as a more accurate and practical biomarker for monitoring therapeutic response [17].

## 4. Conclusions

We present two pediatric cases of cystinuria caused by SLC7A9 mutations, each treated with a different cystine-binding thiol: tiopronin and D-penicillamine. These cases highlight the role of cystine-binding thiol therapy following the failure of conservative measures and underscore the importance of tailoring management to patient response and treatment availability. Despite differing treatment regimens, both patients achieved sustained reductions in urinary cystine excretion and favorable clinical outcomes. While tiopronin is generally preferred due to its more favorable safety profile, our findings support the continued use of D-penicillamine as an effective alternative when tiopronin is unavailable. These cases underscore the importance of medication adherence and patient-centered treatment strategies in the long-term management of cystinuria.

## Figures and Tables

**Table 1:** Demographic and treatment summary of two pediatric patients with cystinuria due to *SLC7A9* mutations. Case 1 was managed with tiopronin, and Case 2 with D-penicillamine. Urinary cystine excretion was effectively controlled in both patients.

	Case 1	Case 2
Gender	Female	Female
Age on Presentation (years)	14	11
Genetic Testing	SLC7A9 (Natera Renasight)	SLC7A9 (Natera Renasight)
Urological surgical interventions for kidney stone management before Cystinuria diagnosis	Two	Four
Urine cystine excretion on diagnosis (mg/24 h) (nl 17–102 umol/24 h) (nl 4–25 mg/24 h) (nl < 48 mg/1.73 m^2^/24 h)	126 mg/24 h or 138 mg/1.73 m^2^/24 h	658 mg/24 h or 807 mg/1.73 m^2^/24 h
Treatment	Tiopronin 11 mg/kg/day in 3 divided doses, titrated to 16 mg/kg/day to keep urinary cystine excretion < 75 mg/day	D-Penicillamine 33 mg/kg/day in 3 divided doses, titrated as needed to keep urinary cystine excretion < 75 mg/day

**Table 2:** Laboratory data of two pediatric patients with cystinuria due to *SLC7A9* mutations. Case 1 was treated with tiopronin and Case 2 with D-penicillamine. Both patients maintained urinary pH within target range and had no proteinuria. For Case 1, supersaturation of cystine (ssCys) and cystine capacity were within target per Litholink analysis. For Case 2, samples were processed through Quest diagnostic, and ssCys and capacity data were not available. Note: ssCys: defined as the ratio of measured cystine concentration to its solubility in urine. Cystine capacity: a measure of the urine’s ability to dissolve or precipitate cystine, expressed in mg/L. Positive values indicate cystine remains in solution, while negative values indicate a propensity for cystine precipitation.

	UV24 (L/day)	Cys24 (mg/day)	SS Cys	Capacity (mg/L)	pH	Urine Protein/Cr (mg/mg)
Goal	>1.5	<75	<0.6	>150	>7.0	<0.2
Case 1	1.79–3.69	72–236	0.1–0.62	43–268	6.7–7.1	<0.2
Case 2	0.4–3.5	130–1498	NA	NA	6.7–7.4	<0.2

## Data Availability

The original contributions presented in this study are included in the article. Further inquires can be directed to the corresponding author.
